# Effects of Pelvic Floor Muscle Training on Female Sexual Function in Women with Stress Urinary Incontinence: A Controlled Observational Study

**DOI:** 10.3390/medicina62071412

**Published:** 2026-07-21

**Authors:** Dimitrios Stamos, Nikolaos Sofikitis, Vaia Sapouna, Aikaterini Maria Astraka, Michail Baltogiannis, Harikleia Maria Sofikiti, Agni Pantou, Ioannis Giannakis, Minas Paschopoulos, Athanasios Zachariou

**Affiliations:** 1 Department of Urology, University of Ioannina, 45110 Ioannina, Greece; dstamos4673@gmail.com (D.S.); nsofikit@uoi.gr (N.S.); agni.pantou@genesisathens.gr (A.P.); uro.giannakis@gmail.com (I.G.); 2 Urology Outpatient Department, Physical Medicine and Rehabilitation Centre Kentavros, 38222 Volos, Greece; vsapouna@uth.gr (V.S.); urologia@otenet.gr (A.M.A.); 3 Young Andrologist Group, Spermatology Laboratory, University of Ioannina, 45110 Ioannina, Greece; dbaltog@outlook.com (M.B.); urology.gram@uhi.gr (H.M.S.); 4 Department of Obstetrics and Gynecology, University of Ioannina, 45110 Ioannina, Greece; mpasxop@uoi.gr

**Keywords:** stress urinary incontinence, pelvic floor muscle training, female sexual dysfunction, Female Sexual Function Index, sexual distress, perineometer, women’s sexual health

## Abstract

*Background and Objectives:* Stress urinary incontinence (SUI) is a common condition among women and is frequently associated with impaired sexual function and increased sexual distress. Pelvic floor muscle training (PFMT) is considered a first-line conservative treatment for SUI; however, its effects on female sexual function remain incompletely understood. The present study aimed to evaluate the impact of PFMT on sexual function, sexual distress, and pelvic floor muscle performance in women with SUI. *Materials and Methods:* This prospective controlled observational study included sexually active women with clinically confirmed SUI and female sexual dysfunction. Participants were allocated either to a control group (Group A) or to a supervised 12-week PFMT program (Group B). Sexual function and distress were assessed using the Female Sexual Function Index (FSFI) and the Female Sexual Distress Scale–Revised (FSDS-R), respectively. Pelvic floor muscle performance was evaluated using Peritron perineometry, including peak vaginal squeeze pressure, endurance, and resting tone. Objective severity of SUI was assessed using the one-hour pad test. *Results:* A total of 102 women completed the study, including 44 in the control group and 58 in the PFMT group. After 12 weeks, women undergoing PFMT demonstrated significant improvements in urinary continence, pelvic floor muscle strength, and sexual function compared with controls (all *p* < 0.01). Mean FSFI total score increased from 21.5 ± 2.5 to 26.6 ± 3.3, while the proportion of women with clinically significant sexual distress decreased from 100% to 58.6%. Peak vaginal squeeze pressure and endurance also improved significantly following PFMT. *Conclusions:* PFMT significantly improves sexual function, reduces sexual distress, and enhances pelvic floor muscle performance in women with SUI. These findings support the integration of PFMT into the comprehensive management of women with SUI and associated sexual dysfunction.

## 1. Introduction

Female sexual function involves several interconnected aspects, including desire, arousal, lubrication, orgasm, satisfaction, and the absence of pain. It is an important part of a woman’s overall quality of life and well-being [1]. Recent research indicates that female sexual dysfunction (FSD) is quite common, with prevalence rates reported between 19% and 50% among women of various ages and backgrounds [2]. The causes of FSD are diverse and include physiological, psychological, and sociocultural factors. Notably, the function of the pelvic floor muscles (PFMs) has been increasingly recognized as a major influence on sexual health [3].

Stress urinary incontinence (SUI) is a prevalent type of urinary incontinence among women, significantly affecting quality of life, emotional health, and intimate partnerships. The effects of SUI go beyond physical symptoms; it can also harm sexual function through fears of urine leakage during sex, feelings of embarrassment, diminished self-esteem, avoidance of intimacy, and lower sexual satisfaction [4]. Additional issues such as coital incontinence, vaginal discomfort, decreased arousal, and negative body image may further impair sexual function [5]. Thus, the link between SUI and sexual health is not purely physical, but also involves psychological and interpersonal factors [5]. Since weakened pelvic floor muscles contribute to SUI, treatments that strengthen these muscles may improve both urinary control and sexual well-being [6].

Pelvic floor muscles are essential for supporting pelvic organs, ensuring urinary continence, and facilitating sexual response. These muscles help maintain vaginal tone, regulate genital blood flow, and produce the rhythmic contractions experienced during orgasm [7]. Problems with these muscles—such as weakness, excessive tension, or poor coordination—can result in symptoms like urinary incontinence, vaginal looseness, and reduced sexual fulfillment. Weakening of the pelvic floor, commonly caused by aging, pregnancy, or childbirth, can diminish vaginal tone and sensation, negatively influencing sexual function [8].

Pelvic floor muscle training (PFMT) is widely recognized as a first-line, non-invasive intervention for urinary incontinence [9]. Beyond its established benefits in continence, increasing attention has been directed toward its potential role in enhancing sexual function [3]. Mechanistically, strengthening the PFMs may improve vaginal tightness, increase local blood circulation, and enhance neuromuscular control, all of which are considered important for sexual arousal and orgasmic capacity [10].

Several clinical and observational studies have reported positive associations between pelvic floor muscle (PFM) strength and multiple domains of female sexual function, including desire, arousal, lubrication, orgasm, and satisfaction [11]. Furthermore, improvements in pelvic floor function following training interventions have been associated with enhanced sexual quality of life and reductions in symptoms such as dyspareunia [6,7,12,13]. However, the existing literature remains heterogeneous, with some studies demonstrating significant improvements in sexual outcomes, while others report limited or no effect, underscoring the need for further investigation into the magnitude and determinants of this relationship [14]. Importantly, most previous studies have evaluated sexual function primarily through the Female Sexual Function Index (FSFI) alone, without concurrently assessing sexual distress, which is an essential criterion for the diagnosis of female sexual dysfunction (FSD). Consequently, relying solely on FSFI scores may provide an incomplete assessment of clinically meaningful sexual dysfunction. Therefore, the present study aimed to evaluate the effect of pelvic floor muscle training on female sexual function in women with stress urinary incontinence, using changes in FSFI scores together with measures of sexual distress as the primary outcomes.

## 2. Materials and Methods

Between May 2024 and April 2026, a total of 183 sexually active women with clinically confirmed SUI were prospectively recruited from the Urology Department of Ioannina University and the Urology Outpatient Clinic of the Kentavros Rehabilitation Centre. SUI was diagnosed based on patient-reported symptoms and clinical evaluation. Women were considered to have SUI if they reported involuntary urine leakage during physical exertion, coughing, sneezing, or other activities that increase intra-abdominal pressure, in the absence of urgency [15]. The diagnosis was confirmed by physical examination, including a positive cough stress test, and supported by objective measures such as pad testing [16]. Additional inclusion criteria were the participants’ willingness to comply with the study protocol and their ability to independently complete voiding diaries and questionnaires.

Eligible participants were required to be over 18 years of age, sexually active, and to present with female sexual dysfunction. Women who reported being sexually inactive were asked to specify the reason and were excluded from further analysis. Participants who declined conservative treatment for SUI—either because they did not perceive a need for treatment or preferred surgical management at a later stage—were allocated to the control group.

Participants were excluded if they had clinical evidence of urgency urinary incontinence, neurogenic bladder dysfunction, urinary retention, or were considered at risk for these conditions. Additional exclusion criteria included previous pelvic floor muscle training, pelvic organ prolapse beyond stage I, prior surgery for urinary incontinence or pelvic organ prolapse, neurological disorders, or active urinary tract infection. The study protocol was approved by the Ethics Committee of Ioannina University and the Kentavros Rehabilitation Centre (approval numbers 57331/24 September 2023 and 25/3 October 2023). Written informed consent was obtained from all participants before enrollment.

Patients’ comprehensive medical history was evaluated, together with detailed general and neurological physical examinations. Urodynamic testing was not performed.

Based on the International Continence Society (ICS) recommendations, uncomplicated stress urinary incontinence (SUI) is diagnosed clinically in women who report involuntary urine leakage during physical activity, exertion, coughing, or sneezing, provided there are no accompanying symptoms of urgency or mixed urinary incontinence, clinically significant pelvic organ prolapse, a history of pelvic floor or incontinence surgery, neurological disorders, voiding dysfunction, recurrent urinary tract infections, or other conditions requiring additional specialized investigation, such as urodynamic assessment. Consequently, routine urodynamic testing is not considered necessary for the initial evaluation of uncomplicated SUI [17]. This recommendation is further supported by evidence demonstrating that clinical assessment alone yields outcomes comparable to those achieved when urodynamic testing is added to the office evaluation in women with uncomplicated, objectively demonstrable SUI [18,19,20].

Urinary leakage was objectively assessed using the one-hour pad test, a simple, non-invasive, and inexpensive method for quantifying urine loss. The test was performed at baseline and after completion of the PFMT program. Participants wore a pre-weighed absorbent pad for one hour, after which the pad was reweighed; the increase in pad weight was recorded as urine loss. A pad weight gains greater than 2 g was considered consistent with incontinence. The test was repeated on three occasions. In the present study, changes in pad weight before and after treatment were used as an objective measure of SUI severity and response to PFMT [21,22].

Because sexual function and quality of life are sensitive domains influenced by stigma, self-administered and patient-reported questionnaires are appropriate assessment tools. The Female Sexual Function Index (FSFI) remains the reference standard for assessing female sexual dysfunction, with level 1 evidence and grade A recommendation [23]. The FSFI is a 19-item self-report questionnaire assessing six domains of female sexual function: desire, arousal, lubrication, orgasm, satisfaction, and pain. Domain and total scores are calculated, with higher scores indicating better sexual function. All participants completed the Greek version of the FSFI, which has been validated [24]. A total FSFI score of ≤26.5 was used to define female sexual dysfunction, according to Wiegel et al. [25].

To identify eligible participants, women with female sexual dysfunction were required to report associated sexual distress. Sexual distress was assessed using the 13-item Female Sexual Distress Scale–Revised (FSDS-R), a validated instrument for distinguishing women with and without sexual dysfunction [26]. The FSDS-R was developed by adding item 13, which assesses distress related to low sexual desire, to the original FSDS. A total FSDS-R score ≥ 11 was considered indicative of clinically significant sexual distress [27].

Pelvic floor muscle training consisted of four supervised biofeedback sessions combined with a home-based exercise program [28]. Before starting the program, all participants received individualized instruction from a trained practitioner, as many women are unable to perform pelvic floor contractions correctly without guidance. Correct contraction was confirmed by vaginal palpation and observation of inward perineal movement [29]. The home exercise program was designed to progressively increase the number of pelvic floor contractions over time. Initially, each exercise set included 5 rapid contractions and 10 sustained contractions, separated by 10 s rest intervals. The program was gradually intensified until participants performed sets of 5 rapid contractions and 20 sustained contractions twice daily.

Trained nurses were responsible for implementing and evaluating the PFMT protocol. During the 12-week intervention period, participants attended weekly clinic visits to monitor technique, adherence, and progress.

Pelvic floor muscle strength was assessed using the Peritron precision perineometer. The device measures the pressure generated during pelvic floor muscle contraction and provides an objective evaluation of muscle strength and endurance. It was also used to support training and monitor progress throughout the exercise program. The Peritron perineometer has previously been shown to be a reliable instrument for clinical research [30]. Maximal voluntary contraction was expressed as peak vaginal squeeze pressure in cmH_2_O, while endurance was recorded as the duration of sustained contraction in seconds. Resting tone was defined as baseline vaginal pressure before voluntary contraction. Women with SUI generally demonstrate lower maximal squeeze pressure and reduced contraction endurance compared with continent women.

A priori sample size calculation was performed for a paired pre–post comparison using FSFI total score as the primary outcome. Assuming a mean improvement of 5 points in total FSFI score, an effect size of 0.425, 80% power, and a two-sided α level of 0.05, the minimum required sample size was estimated to be 46 participants.

Participants were allocated into two groups. Group A served as the control group and included women with SUI who declined treatment. Group B included women with SUI who underwent a structured pelvic floor muscle training program according to the study protocol. Participants in Group A attended monthly visits to confirm that they had not received pharmacological treatment or any other behavioral therapy for SUI during the study period. All participants underwent pad testing and Peritron perineometer assessment, and completed the FSFI and FSDS-R questionnaires at baseline and after completion of the 12-week study period.

Data completeness was checked before statistical analysis. Participants with incomplete baseline or follow-up assessments, missing questionnaire data, or incomplete pelvic floor measurements were excluded from the final analysis. Therefore, analyses were performed using a complete-case approach.

The study was reported in accordance with the Strengthening the Reporting of Observational Studies in Epidemiology (STROBE) guidelines for cohort studies. Statistical analysis was performed using IBM SPSS Statistics, version 29.0 (IBM Corp., Armonk, NY, USA). Normality of data distribution was assessed using the Shapiro–Wilk test. For normally distributed continuous variables, pre–post comparisons were performed using the paired-samples t-test, whereas for non-normally distributed variables, the Wilcoxon signed-rank test was used for pre–post comparisons. A *p*-value < 0.05 was considered statistically significant. Data are presented as mean ± standard deviation (range) or *n* (%), unless otherwise stated.

## 3. Results

A total of 153 women were initially assessed for eligibility in this prospective controlled observational study. Of these, 41 women were excluded before enrollment: 21 were unwilling to participate in the pelvic floor muscle training (PFMT) program, 15 did not wish to complete the FSFI and FSDS-R questionnaires, and 5 refused to undergo the pad test and assessment of stress urinary incontinence (SUI) severity. Therefore, 112 women implemented the study protocol.

Participants were allocated into two groups: the control group (Group A), which included 44 women, and the PFMT group (Group B), which included 68 women with SUI who underwent a structured pelvic floor muscle training program according to the study protocol. During the study period, 10 women in Group B discontinued the PFMT program before completion of the 12-week intervention and were therefore excluded from the final analysis. Consequently, the final study population consisted of 102 women, including 44 in Group A and 58 in Group B. All participants included in the final analysis had complete baseline and follow-up data; therefore, no imputation for missing data was necessary.

Demographic characteristics and baseline clinical data are presented in Table 1. There were no statistically significant differences between groups at baseline (all *p* > 0.05), indicating comparability.

Participants in Groups A had experienced SUI for a mean duration of 6.1 ± 5.0 years and the mean in Group B was 6.7 ± 4.6 years, with a range of 1–10 years. At baseline, the mean FSFI domain scores in Group A were as follows: desire, 2.6 ± 0.3; arousal, 3.5 ± 0.4; lubrication, 3.5 ± 0.3; orgasm, 4.2 ± 0.5; satisfaction, 3.4 ± 0.4; and pain, 3.1 ± 0.4. In Group B, baseline FSFI scores were: desire, 2.8 ± 0.3; arousal, 3.4 ± 0.4; lubrication, 3.4 ± 0.3; orgasm, 4.0 ± 0.5; satisfaction, 3.6 ± 0.4; and pain, 3.1 ± 0.4. Urine leakage during sexual intercourse was reported at a mean frequency of 2 ± 1 episodes in both groups. Values are presented as mean ± standard deviation (Table 2).

Participants completed the PFMT protocol under close supervision and regular follow-up by registered nurses. Changes in incontinence-related outcomes, sexual distress, and sexual function are presented in Table 3.

Twenty-nine women (50%) reported complete resolution of urinary leakage after completing the PFMT program. Following 12 weeks of continuous and successful PFMT, a significant improvement in SUI was observed. This corresponded to an increase of approximately 4.7–5 points in the FSFI total score following PFMT. At the end of treatment, the mean FSFI domain scores were: desire, 4.0 ± 0.6; arousal, 4.6 ± 0.6; lubrication, 4.5 ± 0.4; orgasm, 4.6 ± 0.5; satisfaction, 4.4 ± 0.4; and pain, 4.8 ± 0.5. Urinary leakage episodes during sexual intercourse also decreased significantly after treatment, reaching 0.4 ± 0.5 episodes (*p* < 0.001). No significant changes were observed in the control group in any of the outcome measures (Table 4).

**Figure 1:**
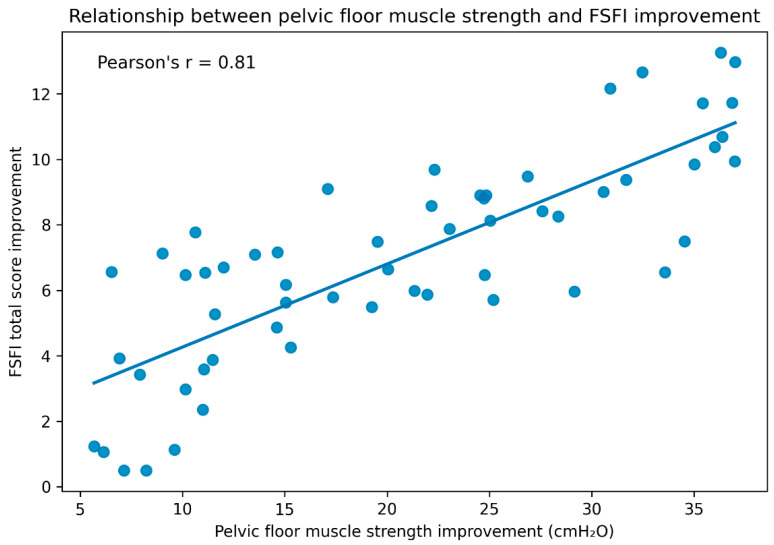
Scatterplot showing the correlation between improvement in FSFI total score and peak vaginal squeeze pressure.

**Figure 2:**
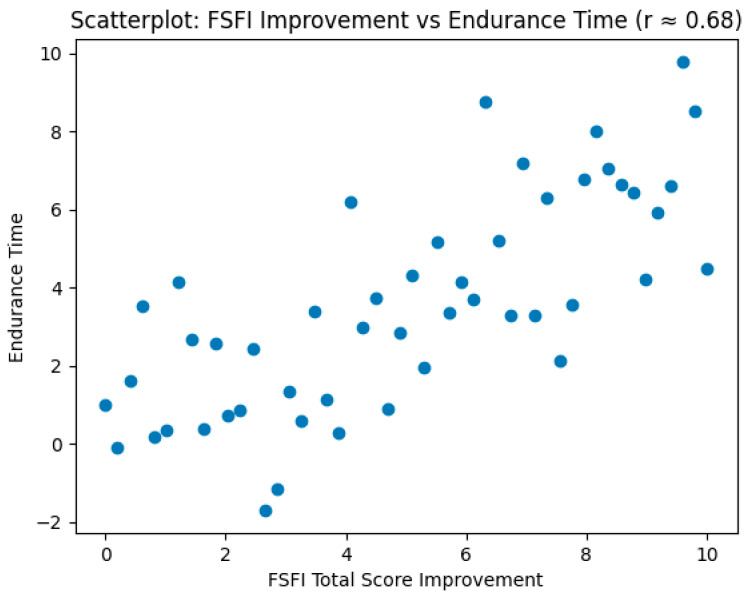
Scatterplot showing the correlation between improvement in FSFI total score and endurance.

A correlation analysis was performed to examine the association between improvement in the FSFI total score and pelvic floor peak pressure (Figure 1). The scatterplot demonstrated a strong positive correlation, with Pearson’s r = 0.81 (*p* < 0.05). A similar analysis was conducted for FSFI total score improvement and endurance time (Figure 2), showing another strong positive association, although of lower magnitude, with Pearson’s r = 0.68 (*p* < 0.05).

## 4. Discussion

The present prospective controlled observational study demonstrates that PFMT is associated with significant improvements in female sexual function in women with SUI. Specifically, a clinically meaningful increase of approximately 5 points in total FSFI score was observed following the 12-week intervention, accompanied by improvements across all sexual function domains.

The observed improvement in sexual function following PFMT can be explained through both physiological and psychosocial mechanisms. From a physiological perspective, pelvic floor muscles contribute directly to the female sexual response, particularly during arousal and orgasm, through rhythmic involuntary contractions, support of the vaginal wall, and modulation of genital sensation [14]. Stronger and better-coordinated pelvic floor muscles may enhance vaginal tone, improve neuromuscular control, and facilitate genital vascular responses, which may explain improvements in FSFI domains such as arousal, orgasm, satisfaction, and pain reported in previous studies and systematic reviews [31,32]. In women with stress urinary incontinence, PFMT may also improve sexual function indirectly by reducing urine leakage, including coital incontinence, which is known to negatively affect desire, comfort, self-image, and sexual satisfaction. Therefore, improved continence may reduce fear of leakage or embarrassment during intercourse, alleviate performance-related anxiety, and enhance sexual confidence, intimacy, and overall sexual quality of life [33]. These combined mechanisms suggest that the benefit of PFMT is not limited to restoration of pelvic floor strength, but also includes improvements in body perception, emotional well-being, and partner-related sexual satisfaction [34].

Importantly, the strong correlation observed in our study between FSFI improvement and peak vaginal squeeze pressure supports the hypothesis that pelvic floor muscle function is a key determinant of sexual health. This relationship has been previously suggested, with stronger pelvic floor muscles contributing to enhanced sexual response and satisfaction [6,35,36]. The slightly lower correlation with endurance further indicates that both strength and sustained contraction capacity play complementary roles in sexual function [13].

The findings indicate that recovery of pelvic floor muscle performance was closely associated with better sexual outcomes. Women demonstrated significant improvements in both objective measures of pelvic floor function and self-reported sexual health. These changes were accompanied by a clinically relevant increase in the overall FSFI score, with benefits observed in every domain of the questionnaire, including sexual desire, arousal, lubrication, orgasm, satisfaction, and pain. At the same time, continence outcomes improved substantially, as evidenced by lower urine loss on the one-hour pad test and a pronounced decline in urine leakage during sexual activity. These findings suggest that enhanced pelvic floor function may improve sexual well-being through multiple mechanisms, including better continence, greater self-confidence, and reduced fear of leakage during intimacy. Correlation analyses further supported this relationship. The magnitude of improvement in sexual function showed a strong positive association with increases in peak vaginal squeeze pressure (r = 0.81), indicating that women who achieved larger gains in pelvic floor muscle strength tended to report greater improvements in sexual function. Improvements in FSFI scores were also positively correlated with longer contraction endurance (r = 0.68), highlighting the contribution of muscular endurance to sexual health. However, the stronger correlation observed for maximal squeeze pressure suggests that muscle strength may have a more prominent influence on female sexual function than endurance within the present cohort.

The significant reduction in sexual distress observed in our study also aligns with prior evidence indicating that PFMT may improve not only physical aspects of sexual function but also psychological well-being. Sexual distress is a critical component of female sexual dysfunction, and its improvement reflects a broader positive impact on quality of life [37,38].

Our results are in agreement with previous clinical studies demonstrating that PFMT improves sexual function in women with SUI. According to Blanco-Ratto et al., pelvic floor exercises alone and in combination with vaginal spheres significantly improved urinary incontinence symptoms, pelvic floor muscle strength, and several domains of sexual function. However, women using vaginal spheres showed greater improvements in sexual desire, arousal, and lubrication, suggesting a potential additional benefit of this adjunctive therapy. Importantly, no significant between-group differences were observed in overall FSFI or incontinence scores, indicating that pelvic floor muscle training itself remains the main therapeutic factor [39]. Citak et al. demonstrated that pelvic floor muscle training (PFMT) was associated with significant improvements in both pelvic floor muscle strength and female sexual function among postpartum women. Participants who regularly performed pelvic floor exercises achieved higher scores in several domains of sexual function, including arousal, lubrication, orgasm, satisfaction, and total FSFI score, compared with women in the control group. Their findings suggest that enhancement of pelvic floor muscle performance may contribute to improved sexual well-being, responsiveness, and overall quality of life [40]. Nazarpour et al. reported that, following a 12-week PFMT intervention, women in both the formal sex education and Kegel exercise groups demonstrated significantly higher arousal scores than those in the control group (3.38 and 3.15 versus 2.77, respectively). In addition, participants performing Kegel exercises achieved significantly greater orgasm and satisfaction scores compared with controls (4.43 and 4.88 versus 3.95 and 4.39, respectively) [32]. Hadizadeh-Talasaz et al. [41], in a meta-analysis evaluating postpartum women, reported that pelvic floor muscle training was associated with improvements in both sexual function and quality of life among primiparous and multiparous women. These benefits may be explained by enhanced pelvic floor muscle strength, increased pelvic support, improved blood circulation, and greater confidence during sexual activity. However, many previous studies did not assess sexual distress, which represents an essential component in the diagnosis and evaluation of female sexual dysfunction [42,43].

On the other hand, Lau et al. reported that although pelvic floor muscle training (PFMT) significantly improved pelvic floor muscle strength, urinary symptoms, and quality of life in women with stress urinary incontinence, no significant improvement was observed in sexual function scores. These findings suggest that female sexual function is multifactorial and may be influenced not only by pelvic floor performance, but also by psychological, relational, and sociocultural factors [12].

The magnitude of FSFI improvement observed in our study is comparable to that reported in recent interventional studies and meta-analyses, which have highlighted clinically meaningful benefits of PFMT, particularly when training is supervised and adheres to structured protocols [14,31,32,34,39,41]. The use of biofeedback and regular follow-up in our protocol may have contributed to the effectiveness of the intervention, as supervision has been shown to enhance adherence and optimize outcomes [44,45].

Additionally, our findings support the concept that improvements in sexual function are not solely attributable to anatomical or physiological changes, but also to psychological and behavioral factors. Women with SUI often experience embarrassment, reduced self-esteem, and avoidance of sexual activity, and improvement in continence may alleviate these barriers, facilitating a more positive sexual experience [5,6,8,46].

The strengths of this study include its prospective design, the use of validated and widely accepted instruments (FSFI and FSDS-R), and the incorporation of objective measures of pelvic floor muscle function using perineometry. The standardized PFMT protocol and close supervision of participants further enhance the reliability of the findings.

However, several limitations should be acknowledged. First, the non-randomized design introduces the possibility of selection bias, as group allocation was based on participants’ treatment preference. Second, the relatively short follow-up period limits the ability to assess the long-term sustainability of the observed improvements. Third, no multivariable adjustment for potential confounders was performed, which may affect the precision of the estimated associations. Additionally, the study relied on self-reported measures of sexual function, which may be subject to reporting bias, although validated questionnaires were used to mitigate this limitation. To reduce measurement bias, all participants were assessed using the same standardized protocol, validated questionnaires, and objective pelvic floor measurements at baseline and follow-up. Selection bias could not be completely eliminated, as allocation to the control group was based on participants’ decision to decline conservative treatment. Because participation in treatment was voluntary, women who declined pelvic floor muscle training may have differed systematically from those who enrolled. Differences in factors such as motivation, expectations regarding treatment efficacy, health-related attitudes, or other psychosocial characteristics could have affected subjective outcome measures, especially the psychological aspects of sexual function captured by the Female Sexual Function Index (FSFI).

The findings of this study are applicable to sexually active women with stress urinary incontinence and coexisting sexual dysfunction, particularly those willing to engage in structured PFMT programs. However, generalizability may be limited in populations with different demographic or clinical characteristics, such as women with severe pelvic organ prolapse, neurological conditions, or those who are sexually inactive. Further studies are needed to confirm these findings across broader and more diverse populations.

## 5. Conclusions

The results of this study reinforce the role of PFMT as a first-line, non-invasive intervention not only for urinary incontinence but also for improving female sexual function. Given its safety, low cost, and accessibility, PFMT should be considered an integral component of the management of women with SUI and associated sexual dysfunction. Pelvic floor muscle training significantly improves sexual function, reduces sexual distress, and enhances pelvic floor muscle performance in women with stress urinary incontinence. These findings highlight the close relationship between pelvic floor function and female sexual health and support the integration of PFMT into routine clinical practice for the comprehensive management of affected women.

Future research should focus on randomized controlled trials with larger sample sizes, standardized intervention protocols, and longer follow-up periods to better define the magnitude and durability of PFMT effects. Additionally, investigating the role of adjunctive therapies, such as biofeedback, digital health tools, and multidisciplinary interventions, may further enhance treatment outcomes.

## Figures and Tables

**Table 1:** Baseline demographic and clinical characteristics of women in the PFMT group (Group B, n = 58) and the control group (Group A, n = 44).

	Control, Group A *n* = 44	PFMT, Group B *n* = 58	*p*
Age, years	48.3 ± 10.4	49.7 ± 11.1	*p* > 0.05
Parity	2.4 ± 0.9	2.2 ± 1.1	*p* > 0.05
Vaginal delivery	31 (70.4%)	39 (67.2%)	*p* > 0.05
Cesarian section	4 (9.0%)	5 (8.62%)	*p* > 0.05
Body mass index, Kg/m^2^	25.1 ± 4.1	24.2 ± 3.8	*p* > 0.05
Hypertension	6 (13.6%)	9 (15.5%)	*p* > 0.05
Diabetes	2 (4.5%)	3 (5.1%)	*p* > 0.05
Postmenopausal status	14 (31.8%)	21 (36.2%)	*p* > 0.05
Pad test (1 h, g)	5.9 ± 2.1	6.2 ± 2.4	*p* > 0.05
FSFI Total	20.3 ± 2.3	21.5 ± 2.5	*p* > 0.05
FSDS-R ≥ 11	44 (100%)	58 (100%)	*p* > 0.05
Perineometer Peritron			
Peak pressure, cmH_2_O	18.27 ± 10.6	19.12 ± 10.1	*p* > 0.05
Endurance	4.3 ± 2.1 s	4.5 ± 2.3 s	*p* > 0.05
Resting tone, cmH_2_O	10.45 ± 7.49	11.04 ± 7.99	*p* > 0.05

Values are mean ± SD or *n* (%). Abbreviations: PFMT, Pelvic Floor Muscle Training; FSFI, Female Sexual Function Index; FSDS-R, Female Sexual Distress Scale Revised.

**Table 2:** Baseline FSFI domain scores in the study groups.

FSFI Domain	Control Group (Group A, *n* = 44)	PFMT Group (Group B, *n* = 58)	*p* Value
Desire	2.6 ± 0.3	2.8 ± 0.3	>0.05
Arousal	3.5 ± 0.4	3.4 ± 0.4	>0.05
Lubrication	3.5 ± 0.3	3.4 ± 0.3	>0.05
Orgasm	4.2 ± 0.5	4.0 ± 0.5	>0.05
Satisfaction	3.4 ± 0.4	3.6 ± 0.4	>0.05
Pain	3.1 ± 0.4	3.1 ± 0.4	>0.05

Values are mean ± SD. Abbreviations: PFMT, Pelvic Floor Muscle Training; FSFI, Female Sexual Function Index.

**Table 3:** Changes in incontinence-related outcomes, sexual distress, and sexual function after the 12-week PFMT protocol in Group B, compared with the corresponding parameters in Group A (control group).

	Control, Group A *n* = 44	PFMT, Group B *n* = 58	*p*
Pad test (1 h, g)	4.7 ± 2.0	2.9 ± 1.4	*p* < 0.01
FSFI Total	20.9 ± 2.1	26.6 ± 3.3	*p* < 0.01
FSDS-R ≥ 11	44 (100%)	34 (58.6%)	*p* < 0.01
Perineometer Peritron			
Peak pressure	18.27 ± 10.6 cmH_2_O	41.42 ± 15.1 cmH_2_O	*p* < 0.01
Endurance	4.3 ± 2.1 s	9.7 ± 4.3 s	*p* < 0.01
Resting tone	10.45 ± 7.49 cmH_2_O	13.8 ± 9.58 cmH_2_O	*p* > 0.05

Values are mean ± SD or *n* (%). Abbreviations: PFMT, Pelvic Floor Muscle Training; FSFI, Female Sexual Function Index; FSDS-R, Female Sexual Distress Scale Revised.

**Table 4:** Post-treatment FSFI domain scores in the study groups.

FSFI Domain	Control Group (Group A, *n* = 44)	PFMT Group (Group B, *n* = 58)	*p* Value
Desire	2.8 ± 0.3	4.0 ± 0.6	<0.05
Arousal	3.6 ± 0.4	4.6 ± 0.6	<0.05
Lubrication	3.6 ± 0.3	4.5 ± 0.4	<0.05
Orgasm	4.1 ± 0.5	4.6 ± 0.5	<0.05
Satisfaction	3.5 ± 0.4	4.4 ± 0.4	<0.05
Pain	3.2 ± 0.4	4.8 ± 0.5	<0.05

Values are mean ± SD. Abbreviations: PFMT, Pelvic Floor Muscle Training; FSFI, Female Sexual Function Index.

## Data Availability

The authors declare that they have followed their center’s protocols on the publication of patient data. All data analyzed during the current study are available from the corresponding author on reasonable request. The authors have reviewed and edited the content and take full responsibility for the final version of this publication.

## Abbreviations

The following abbreviations are used in this manuscript:

ICS	International Continence Society
FSD	Female Sexual Dysfunction
FSDS	Female Sexual Distress Scale
FSDS-R	Female Sexual Distress Scale–Revised
FSFI	Female Sexual Function Index
PFM	Pelvic Floor Muscle
PFMT	Pelvic floor muscle training
SUI	Stress urinary incontinence

## References

[1] Rosen R., Brown C., Heiman J., Leiblum S., Meston C., Shabsigh R., Ferguson D., D’Agostino R. (2000). The Female Sexual Function Index (FSFI): A multidimensional self-report instrument for the assessment of female sexual function. J. Sex Marital Ther..

[2] Shifren J.L., Monz B.U., Russo P.A., Segreti A., Johannes C.B. (2008). Sexual problems and distress in United States women: Prevalence and correlates. Obstet. Gynecol..

[3] Stamos D., Sapouna V., Astraka K.M., Thanopoulou S., Giannakis I., Pantou A., Baltogiannis D., Paschopoulos M., Sofikitis N., Zachariou A. (2025). Female Sexual Function and Pelvic Floor Muscle Training: A Narrative Review. Cureus.

[4] Salonia A., Zanni G., Nappi R.E., Briganti A., Dehò F., Fabbri F., Colombo R., Guazzoni G., Di Girolamo V., Rigatti P. (2004). Sexual dysfunction is common in women with lower urinary tract symptoms and urinary incontinence: Results of a cross-sectional study. Eur. Urol..

[5] Duralde E.R., Rowen T.S. (2017). Urinary Incontinence and Associated Female Sexual Dysfunction. Sex. Med. Rev..

[6] Zahariou A.G., Karamouti M.V., Papaioannou P.D. (2008). Pelvic floor muscle training improves sexual function of women with stress urinary incontinence. Int. Urogynecol. J..

[7] Faucher S., Déry-Rouleau G., Bardin M., Morin M. (2024). Investigating the role of the pelvic floor muscles in sexual function and sexual response: A systematic review and meta-analysis. J. Sex. Med..

[8] Frigerio M., Barba M., Cola A., Braga A., Celardo A., Munno G.M., Schettino M.T., Vagnetti P., De Simone F., Di Lucia A. (2022). Quality of Life, Psychological Wellbeing, and Sexuality in Women with Urinary Incontinence—Where Are We Now: A Narrative Review. Medicina.

[9] Bø K. (2004). Urinary incontinence, pelvic floor dysfunction, exercise and sport. Sports Med..

[10] García-Laria R., Alonso-Calvete A., Justo-Cousiño L., Da Cuña-Carrera I., Soto-González M. (2025). Effects of pelvic floor muscle training on sexual function of postmenopausal women: A systematic review and meta-analysis. Sex. Med..

[11] Hwang U.J., Lee M.S. (2023). Relationship between female sexual function, vaginal volume, vaginal resting tone, and pelvic floor muscle strength in women with stress urinary incontinence. Obstet. Gynecol. Sci..

[12] Lau H.H., Su T.H., Hwang J.C. (2024). Impact of pelvic floor muscle training on sexual function in women affected by stress urinary incontinence. Sex. Med..

[13] Pasqualotto L., Riccetto C., Biella A.F., Marques J., Pereira L.C., Alves F.K., Botelho S. (2022). Impact of pelvic floor muscle strength on female sexual function: Retrospective multicentric cross-sectional study. Int. Urogynecol. J..

[14] Jorge C.H., Bø K., Catai C.C., Brito L.G.O., Driusso P., Tennfjord M.K. (2024). Pelvic floor muscle training as treatment for female sexual dysfunction: A systematic review and meta-analysis. Am. J. Obstet. Gynecol..

[15] Lukacz E.S., Santiago-Lastra Y., Albo M.E., Brubaker L. (2017). Urinary Incontinence in Women: A Review. JAMA.

[16] Price D.M., Noblett K. (2012). Comparison of the cough stress test and 24-h pad test in the assessment of stress urinary incontinence. Int. Urogynecol. J..

[17] National Institute for Health and Care Excellence (NICE) (2019). Urinary Incontinence and Pelvic Organ Prolapse in Women: Management.

[18] Nager C.W., Brubaker L., Litman H.J., Zyczynski H.M., Varner R.E., Amundsen C., Sirls L.T., Norton P.A., Arisco A.M., Chai T.C. (2012). A randomized trial of urodynamic testing before stress-incontinence surgery. N. Engl. J. Med..

[19] Finazzi-Agrò E., Serati M., Salvatore S., Del Popolo G. (2013). Comments on “A randomized trial of urodynamic testing before stress-incontinence surgery”. Neurourol. Urodyn..

[20] Nager C.W., Urinary Incontinence Treatment Network (2013). Re: Comments on “A randomized trial of urodynamic testing before stress-incontinence surgery”. Neurourol. Urodyn..

[21] Ferreira C.H., Bø K. (2015). The Pad Test for urinary incontinence in women. J. Physiother..

[22] Wu W.Y., Sheu B.C., Lin H.H. (2008). Twenty-minute pad test: Comparison of infusion of 250 mL of water with strong-desire amount in the bladder in women with stress urinary incontinence. Eur. J. Obstet. Gynecol. Reprod. Biol..

[23] Hatzichristou D., Kirana P.S., Banner L., Althof S.E., Lonnee-Hoffmann R.A., Dennerstein L., Rosen R.C. (2016). Diagnostic Evaluation of Sexual Dysfunctions in Men and Women and the use of Symptom Scales and Questionnaires. J. Sex. Med..

[24] Zachariou A., Filiponi M., Kirana P.S. (2017). Translation and validation of the Greek version of the female sexual function index questionnaire. Int. J. Impot. Res..

[25] Wiegel M., Meston C., Rosen R. (2005). The female sexual function index (FSFI): Cross-validation and development of clinical cutoff scores. J. Sex Marital Ther..

[26] Derogatis L., Clayton A., Lewis-D’Agostino D., Wunderlich G., Fu Y. (2008). Validation of the female sexual distress scale-revised for assessing distress in women with hypoactive sexual desire disorder. J. Sex. Med..

[27] Santos-Iglesias P., Mohamed B., Danko A., Walker L.M. (2018). Psychometric Validation of the Female Sexual Distress Scale in Male Samples. Arch. Sex. Behav..

[28] Dumoulin C., Cacciari L.P., Hay-Smith E.J.C. (2018). Pelvic floor muscle training versus no treatment, or inactive control treatments, for urinary incontinence in women. Cochrane Database Syst. Rev..

[29] Mota R.L. (2017). Female urinary incontinence and sexuality. Int. Braz. J. Urol..

[30] Frawley H. (2006). Pelvic floor muscle strength testing. Aust. J. Physiother..

[31] Pourkhiz Z., Mohammad-Alizadeh-Charandabi S., Mirghafourvand M., Haj-Ebrahimi S., Ghaderi F. (2017). Effect of pelvic floor muscle training on female sexual function during pregnancy and postpartum: A randomized controlled trial. Iran. Red Crescent Med. J..

[32] Nazarpour S., Simbar M., Ramezani Tehrani F., Alavi Majd H. (2017). Effects of sex education and Kegel exercises on the sexual function of postmenopausal women: A randomized clinical trial. J. Sex. Med..

[33] Basgol S., Oskay U. (2016). Examining the effectiveness of home-based pelvic floor muscle training in treating sexual dysfunction in women. Int. J. Caring Sci..

[34] Franco M.M., Pena C.C., de Freitas L.M., Antônio F.I., Lara L.A., Ferreira C.H.J. (2021). Pelvic floor muscle training effect in sexual function in postmenopausal women: A randomized controlled trial. J. Sex. Med..

[35] Lowenstein L., Gruenwald I., Gartman I., Vardi Y. (2010). Can stronger pelvic muscle floor improve sexual function?. Int. Urogynecol. J..

[36] Martinez C.S., Ferreira F.V., Castro A.A., Gomide L.B. (2014). Women with greater pelvic floor muscle strength have better sexual function. Acta Obstet. Gynecol. Scand..

[37] Zachariou A., Zikopoulos A., Sapouna V., Skentou C., Kaltsas A., Giannakis I., Zachariou D., Dimitriadis F., Mamoulakis C., Mai D.B.T. (2024). Supervised pelvic floor muscle training improves sexual function and diminishes sexual distress in women with relapsing-remitting multiple sclerosis: A randomised controlled study. J. Pers. Med..

[38] Martínez-Galiano J.M., Peinado-Molina R.A., Martínez-Vazquez S., Hita-Contreras F., Delgado-Rodríguez M., Hernández-Martínez A. (2024). Influence of pelvic floor disorders on sexuality in women. Int. J. Gynecol. Obstet..

[39] Blanco-Ratto L., Ramirez-Garcia I., Kauffmann S., Ortiz C.N., Farrés M.G. (2025). Effect of vaginal spheres and pelvic floor exercises on female sexual function in women with urinary incontinence: A randomized controlled trial. Int. Urogynecol. J..

[40] Citak N., Cam C., Arslan H., Karateke A., Tug N., Ayaz R., Celik C. (2010). Postpartum sexual function of women and the effects of early pelvic floor muscle exercises. Acta Obstet. Gynecol. Scand..

[41] Hadizadeh-Talasaz Z., Sadeghi R., Khadivzadeh T. (2019). Effect of pelvic floor muscle training on postpartum sexual function and quality of life: A systematic review and meta-analysis of clinical trials. Taiwan J. Obstet. Gynecol..

[42] Golmakani N., Zare Z., Khadem N., Shareh H., Shakeri M.T. (2015). The effect of pelvic floor muscle exercises program on sexual self-efficacy in primiparous women after delivery. Iran. J. Nurs. Midwifery Res..

[43] Wang Y., Chen W., Li W. (2022). To compare the effects of two pelvic floor muscle treatments on quality of life and sexual function in female patients with urinary incontinence. Sex. Med..

[44] Baumann F.T., Reimer N., Gockeln T., Reike A., Hallek M., Ricci C., Zopf E.M., Schmid D., Taaffe D., Newton R.U. (2022). Supervised pelvic floor muscle exercise is more effective than unsupervised pelvic floor muscle exercise at improving urinary incontinence in prostate cancer patients following radical prostatectomy: A systematic review and meta-analysis. Disabil. Rehabil..

[45] Kharaji G., ShahAli S., Ebrahimi-Takamjani I., Sarrafzadeh J., Sanaei F., Shanbehzadeh S. (2023). Supervised versus unsupervised pelvic floor muscle training in the treatment of women with urinary incontinence: A systematic review and meta-analysis. Int. Urogynecol. J..

[46] Moradinasab S., Iravani M., Mousavi P., Cheraghian B., Molavi S. (2023). Effect of cognitive-behavioral therapy on sexual self-esteem and sexual function of reproductive-aged women suffering from urinary incontinence. Int. Urogynecol. J..

